# Association of CYP3A5 Genetic Variants With Elevated Breast Cancer Risk: A Case–Control and In Silico Analysis

**DOI:** 10.1155/tbj/9977157

**Published:** 2026-09-30

**Authors:** Sm Faysal Bellah

**Affiliations:** ^1^ Department of Pharmacy Pabna University of Science and Technology Pabna 6600 Bangladesh pust.ac.bd

**Keywords:** Bangladeshi women, breast cancer, *CYP3A5*, immune-cell infiltration and survival, polymorphisms

## Abstract

**Background:**

Cytochrome P450 3A5 (CYP3A5) is a key xenobiotic‐metabolizing enzyme that also participates in the biotransformation of endogenous substrates. Functional genetic variation within CYP3A5 can alter enzyme activity and may consequently modify individual susceptibility to malignancy. The contribution of CYP3A5 genetic variation to breast cancer risk, as well as its potential relevance to tumor biology, remains inadequately characterized in the Bangladeshi population.

**Objective:**

The current study investigated the relationship of CYP3A5∗3 (rs776746) polymorphism with the susceptibility of breast cancer and explored its relationship with immune infiltration and survival outcomes.

**Methods:**

The study was conducted as a case–control study including 170 breast cancer patients and 170 age‐matched healthy individuals. CYP3A5∗3 (rs776746) genotypes were determined using polymerase chain reaction–restriction fragment length polymorphism (PCR–RFLP) analysis. Genetic associations were calculated using odds ratios (ORs) with 95% confidence intervals (CIs). Complementary computational analyses evaluated the association of CYP3A5 expression with tumor immune‐cell infiltration using TIMER2.0 and examined its prognostic relevance through survival data from TCGA accessed via UALCAN.

**Results:**

CYP3A5∗3 genotype frequencies differed significantly between cases and controls. Compared with the wild‐type genotype (∗1/∗1), the heterozygote (∗1/∗3) genotype (OR = 2.57; *p* = 0.0016), mutant homozygote (∗3/∗3) (OR = 1.78; *p* = 0.0376), and combined variant genotypes (∗1/∗3 + ∗3/∗3) (OR = 2.11; *p* = 0.0010) were associated with increased breast cancer risk. The CYP3A5∗3 allele was more prevalent in cases than controls (36% vs. 25%; OR = 1.75; *p* = 0.0009). In silico analysis showed that CYP3A5 expression was positively correlated with immune‐cell infiltration of several immune cells. The elevated expression of CYP3A5 was linked to poorer survival in selected clinical subgroups.

**Conclusion:**

The findings indicate that the CYP3A5∗3 polymorphism may contribute to genetic susceptibility to breast cancer among Bangladeshi women. Additionally, CYP3A5 expression shows a relationship with immune infiltration and may have subgroup‐specific prognostic relevance in breast cancer.

## 1. Introduction

Breast cancer imposes a substantial global health burden and represents a leading cancer diagnosis among women worldwide [1]. It also contributes substantially to cancer‐related mortality, accounting for a significant proportion of deaths in both developed and developing countries [2]. Although global advances in screening and therapeutic strategies have been achieved, the global burden of breast cancer continues to increase, with low‐ and middle‐income countries disproportionately affected by delayed diagnosis and inadequate access to cancer care. In Bangladesh, breast cancer constitutes the predominant malignancy among women, underscoring the need to better characterize its genetic, environmental, and clinical determinants to inform effective prevention and early‐detection strategies [3].

Breast cancer arises through a multifaceted interplay of inherited genetic susceptibility, hormonal influences, environmental exposures, and behavioral factors, with their combined effects shaping individual disease risk [4]. Polymorphisms within these pathways can alter the biotransformation of endogenous substrates, xenobiotics, and carcinogenic compounds, thereby potentially modifying exposure to biologically active metabolites and influencing susceptibility to malignancy and treatment outcomes. Genetic variation in drug‐metabolizing enzymes has emerged as an important determinant of interindividual cancer risk and therapeutic response [5].

The cytochrome P450 (CYP) superfamily constitutes a major component of Phase I metabolism pathways, catalyzing the metabolism of diverse xenobiotics, environmental carcinogens, and pharmacological compounds. Within this family, CYP3A5 contributes to the biotransformation of numerous clinically and therapeutically important compounds, including steroid hormones and anticancer drugs [6]. A common functional polymorphism in the CYP3A5 gene, known as CYP3A5∗3, results from a single nucleotide substitution (A > G) that creates an alternative splicing site, leading to the production of a truncated, nonfunctional protein [7]. Individuals carrying the CYP3A5∗3/∗3 genotype typically exhibit markedly reduced or absent CYP3A5 enzyme expression, whereas carriers of the CYP3A5∗1 allele retain functional enzyme activity [8].

Variations in CYP3A5 expression may influence the metabolic processing of carcinogens and endogenous hormones, potentially affecting breast cancer susceptibility. In addition, CYP3A5 polymorphisms have been implicated in interindividual variability in response to endocrine‐based therapies such as tamoxifen, further highlighting their clinical relevance [9]. However, epidemiological studies investigating the association between CYP3A5 genetic variants and breast cancer risk have produced inconsistent findings across different populations, suggesting potential ethnic and environmental heterogeneity [10].

Although several genes, including BRCA1, BRCA2, TP53, and other metabolic and hormone‐related genes, have been strongly associated with breast cancer risk, evidence regarding CYP3A5 remains limited and population‐dependent [11]. Notably, allele frequencies of CYP3A5∗3 vary significantly across ethnic groups, which may contribute to differences in cancer susceptibility profiles worldwide. This variability underscores the importance of conducting population‐specific studies, particularly in underrepresented regions such as South Asia, where genetic data remain limited.

Given the increasing burden of breast cancer in Bangladesh and the limited genomic evidence available from this population, investigating CYP3A5 polymorphisms may provide valuable insights into population‐specific genetic susceptibility. In addition to evaluating the relationship between CYP3A5 genetic variants and breast cancer susceptibility, this study further explores the potential clinical significance of CYP3A5 expression through immune infiltration patterns and survival analysis. These integrated genetic and molecular findings may improve the understanding of CYP3A5‐mediated mechanisms in breast cancer development and contribute to the identification of potential biomarkers for risk assessment.

## 2. Materials and Methods

### 2.1. Study Population and Ethical Considerations

This study enrolled 170 histopathologically identified breast cancer cases and 170 age‐matched controls among Bangladeshi women. Participants were unrelated and recruited from the same geographic area to minimize potential selection and population‐stratification bias. Patients were enrolled from Ahsania Mission Cancer Hospital, Khulna Medical College Hospital, NICRH, and Dhaka Medical College Hospital. Control participants were screened clinically, with mammographic assessment performed where appropriate, to exclude previously undiagnosed breast malignancy. Before enrollment, participants were adequately informed regarding the study goals, processes, and implications and provided written informed consent. Participation was voluntary, and participants retained the right to withdraw without penalty. The study protocol was approved by the Ethical Review Boards of the participating institutions (Approval No. BRCA‐2023‐32) and was conducted in accordance with the principles of the Declaration of Helsinki and its subsequent amendments [12].

### 2.2. Collection of Blood Sample and Isolation of DNA

The case–control participants were enrolled after receiving detailed counseling and a clear description of the study objectives and procedures. After obtaining written informed consent, 3 mL of peripheral venous blood was drawn from each participant into sterile tubes containing EDTA‐Na_2_ as an anticoagulant. The specimens were preserved at −80°C until molecular analysis, after which genomic DNA was purified from whole blood using a validated extraction protocol [13]. DNA quality was assessed spectrophotometrically, with OD260/OD280 ratios between 1.6 and 1.9, indicating acceptable purity for downstream analysis.

### 2.3. Polymerase Chain Reaction–Restriction Fragment Length Polymorphism (PCR–RFLP) Genotyping of CYP3A5 rs776746

The CYP3A5∗3 (rs776746) variant was genotyped by PCR–RFLP. A locus‐specific PCR assay was used to amplify the target genomic region, followed by restriction digestion of the amplified product with the appropriate endonuclease [14]. The forward primer is 5′‐CCTGCCTTCAATTTTTCACT‐3′, and the reverse primer is 5′‐GGTCCAAACAGGGAAGAGGT‐3′. PCR amplification conditions were as follows: PCR was performed in a final reaction volume of 25 μL containing genomic DNA (50–70 ng/μL), 5× GoTaq reaction buffer, MgCl_2_, dNTPs, forward and reverse primers, GoTaq DNA polymerase, and nuclease‐free water. Amplification was initiated with denaturation at 95°C for 5 min, followed by 35 cycles consisting of denaturation at 95°C for 1 min, primer annealing at 66°C for 1 min, and extension at 72°C for 1 min. A final extension step was performed at 72°C for 10 min.

### 2.4. PCR Product Verification, Restriction Digestion, and Quality Control

PCR amplification was verified by electrophoresis on a 10% polyacrylamide gel. For allelic discrimination, 20 μL of each PCR amplicon was subjected to overnight digestion with 2 U of *Rsa*I at 37°C following the manufacturer’s recommended conditions and the previously described protocol [14]. The resulting restriction fragments were resolved by electrophoresis on a 10% polyacrylamide gel prepared in 1 × TBE buffer and used to assign CYP3A5 genotypes according to the expected banding profiles. As an internal quality‐control measure, 30% of samples were randomly selected and independently regenotyped. Genotype concordance was assessed between the initial and repeat analyses. A post hoc power analysis was additionally calculated to evaluate the statistical power of the observed genetic associations.

### 2.5. CYP3A5 Expression and Immune‐Cell Infiltration Analysis in Breast Cancer

The relationship between CYP3A5 expression and the breast cancer immune microenvironment was investigated using TIMER2.0 (https://timer.cistrome.org/), an interactive platform that estimates immune‐cell infiltration from TCGA transcriptomic data. CYP3A5 expression profiles in breast cancer were examined using the Gene_DE module, which enables comparison of gene expression between tumor and nonmalignant tissues. The association between CYP3A5 transcript abundance and the estimated infiltration levels of selected immune‐cell populations was subsequently assessed. Analyses included CD4^+^ and CD8^+^ T cells, macrophages, neutrophils, mast cells, endothelial cells, hematopoietic cells, and cancer‐associated fibroblasts. These analyses were performed to characterize the potential relationship between CYP3A5 expression and the immune contexture of breast tumors [15, 16].

### 2.6. Survival Analysis of CYP3A5 Expression in Breast Cancer

The prognostic significance of CYP3A5 expression was investigated using the UALCAN tool (https://ualcan.path.uab.edu/) with survival data derived from the TCGA breast cancer cohort. Patients were stratified according to CYP3A5 expression levels and relevant clinicodemographic and tumor characteristics, including race, sex, breast cancer subtype, and menopausal status. Kaplan–Meier survival analyses were used to compare overall survival between expression‐defined subgroups. Statistical significance was determined using the log‐rank test. This analysis was performed to explore whether CYP3A5 transcript abundance was associated with survival heterogeneity across clinically defined breast cancer subgroups [17, 18].

### 2.7. Statistical Analysis

Genotype and allele distributions were summarized using proportions and evaluated between breast cancer cases and controls. The association of CYP3A5 genetic variants with breast cancer susceptibility was quantified using logistic regression, with effect estimates reported as odds ratios (ORs) and corresponding 95% confidence intervals (CIs). All statistical analyses were conducted using IBM SPSS Statistics (IBM Corp., Chicago, IL, USA). *p* value < 0.05 was taken as the statistically significant results. For graphical presentation, the statistical significance value was denoted as follows: ns, not significant;  ^∗^ *p* < 0.05;  ^∗∗^ *p* < 0.01; and  ^∗∗∗^ *p* < 0.001.

## 3. Results

### 3.1. Sociodemographic Characteristics and Association of Lifestyle Factors With CYP3A5 Polymorphism Among Study Participants

The sociodemographic, clinical, and genetic profiling of the study population reveals a multifaceted interplay of risk determinants, as illustrated across Figure 1A–H. Religiously, Muslims predominated in both groups (86% patients vs. 79% controls), with Hindus underrepresented among patients (11% vs. 21% controls) and the “Others” category exclusive to the patient group (Figure 1A), hinting at possible lifestyle or healthcare‐seeking behavioral variations. Educational disparities were pronounced, with 61% of patients being illiterate compared to 37% of controls, while higher literacy levels (SSC and HSC) were more frequent among healthy individuals, underscoring education’s protective role (Figure 1B). Geographically, rural residence was slightly overrepresented among cases (65% vs. 61% controls), whereas all participants were indigenous Bangladeshis, ensuring genetic homogeneity. Age‐wise, the 36–45‐year cohort exhibited the highest polymorphism susceptibility (Figure 1C), aligning with known breast cancer risk escalation with advancing age. Breastfeeding patterns (Figure 1D) showed that 71% of subjects had sufficient lactation, yet polymorphism prevalence was strikingly higher in insufficient (100%) and no‐breastfeeding (100%) groups compared to sufficient (80%) or medium (60%) categories (Figure 1E), suggesting a genetic or protective link between adequate breastfeeding and reduced polymorphic variation. Treatment‐wise, allopathic therapy correlated with better health outcomes (74% healthy) than combined homeopathic–allopathic approaches (26% healthy) (Figure 1F). Personal history factors further stratified risk: diabetes, infectious diseases, tobacco–betel leaf use, abortion, and positive family history were associated with elevated polymorphism rates (100%, 66%, 84%, 84%, and 76%, respectively), while childbearing status showed 79% polymorphism among those with children versus 100% among childless individuals (Figure 1G–H), indicating complex reproductive–genetic interactions. Collectively, these findings advocate for integrated public health strategies addressing literacy, rural healthcare access, breastfeeding support, and lifestyle modifications, alongside genetic surveillance, to mitigate breast cancer burden in Bangladesh.

**FIGURE 1:**
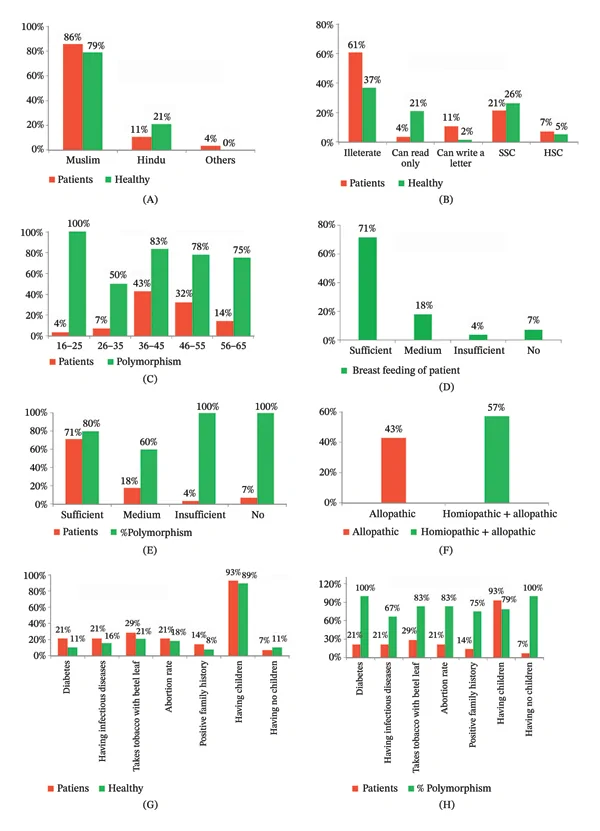
Sociodemographic, clinical, and lifestyle characteristics of study participants and their association with CYP3A5∗3 polymorphism. (A) Distribution of participants according to religion. (B) Comparison of educational status between cases and controls. (C) Age‐wise distribution of patients showing the percentage of CYP3A53 polymorphism. (D) Breastfeeding status among patients. (E) Association between breastfeeding patterns and the percentage of CYP3A53 polymorphism. (F) Distribution of treatment modalities and their relationship with health outcomes. (G) Clinical and personal history of patients in relation to polymorphism frequency. (H) Overall correlation of patients with the observed percentage of CYP3A53 polymorphism across study variables.

### 3.2. CYP3A5 Genotyping and PCR–RFLP Analysis

Genomic DNA from 170 breast cancer cases and 170 healthy individual controls was successfully analyzed to characterize the CYP3A5∗3 polymorphism. The extracted DNA showed acceptable purity (OD260/OD280 ratio: 1.6–1.9). PCR amplification generated the expected 196‐bp CYP3A5 fragment (Figure 2A). Following digestion with the restriction enzyme *Rsa*I, genotype patterns were identified by fragment analysis. Distinct restriction‐fragment patterns were observed for the CYP3A5 genotypes. The wild‐type homozygous genotype (∗1/∗1) produced 102‐, 74‐, and 20‐bp fragments; the heterozygous (HE; ∗1/∗3) genotype yielded 102‐, 94‐, 74‐, and 20‐bp fragments; whereas the mutant homozygous (MH; ∗3/∗3) genotype generated 102‐ and 94‐bp fragments (Figure 2B). These banding patterns were used for CYP3A5 genotype classification among study participants.

**FIGURE 2:**
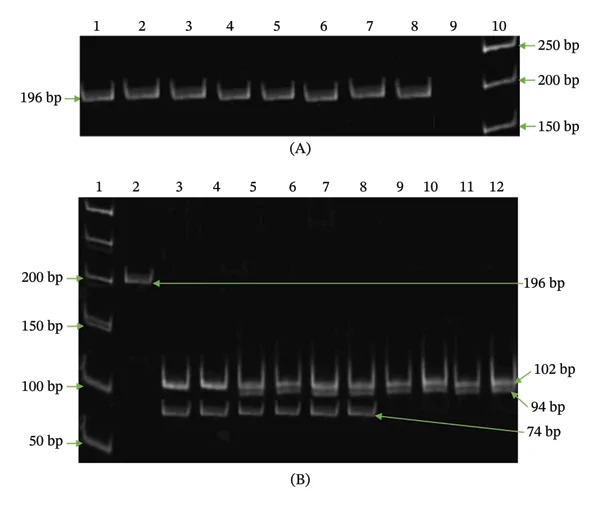
PCR amplification and restriction fragment profiles of the CYP3A5∗3 polymorphism. (A) Representative electrophoretic image of the 196‐bp *CYP3A5*3 PCR product resolved on a 10% polyacrylamide gel. Lanes 1–8 represent participant samples; Lane 9 contains the no‐template control (H_2_O), and Lane 10 contains the molecular size marker. (B) Representative *Rsa*I‐digested PCR products demonstrating genotype‐specific restriction patterns. Lane 1, molecular size marker; Lane 2, undigested 196‐bp PCR product; Lanes 3–4, ∗3/∗3 homozygous variant genotype (102‐, 74‐, and 20‐bp fragments); Lanes 5–8, ∗1/∗3 heterozygous genotype (102‐, 94‐, 74‐, and 20‐bp fragments); Lanes 9–12, ∗1/∗1 wild‐type genotype (102‐ and 94‐bp fragments). All restriction fragments were resolved on a 10% polyacrylamide gel.

### 3.3. CYP3A5∗3 Genetic Polymorphism and Breast Cancer Susceptibility

The potential relationship between the CYP3A5∗3 polymorphism and risk of breast cancer was evaluated by comparing genotype and allele distributions between 170 breast cancer patients and 170 healthy individuals. CYP3A5 genotype frequencies differed significantly in cases and in controls (Table 1). The wild‐type homozygous genotype (∗1/∗1) was more frequent among controls (69%) than breast cancer patients (51%) and was considered the reference genotype. In contrast, the HE genotype (∗1/∗3) occurred in 25% of cases and 13% of controls and was significantly associated with increased breast cancer susceptibility (OR = 2.5674; 95% CI: 1.4293–4.6118; *p* = 0.0016). Similarly, the MH variant (∗3/∗3) revealed a significantly greater susceptibility to breast cancer (OR = 1.7786; 95% CI: 1.0336–3.0607; *p* = 0.0376) as compared to the wild‐type variant. Furthermore, combined analysis of variant genotypes (∗1/∗3 + ∗3/3) revealed a significant association with elevated breast cancer susceptibility (OR = 2.1061; 95% CI: 1.3531–3.2781; *p* = 0.0010), suggesting that carriers of the CYP3A53 allele may have increased genetic predisposition toward breast cancer development.

**TABLE 1:** Comparison of CYP3A5 genotype and allele frequencies between breast cancer patients and healthy controls.

Gene	Genotypes/allele	Cases, *n*, (%)	Controls, *n*, (%)	OR	95% CI	*p* values
CYP3A5	∗1/∗1 (NH)	87 (51)	117 (69)	1.00	—	—
	∗1/∗3 (HE)	42 (25)	22 (13)	2.5674	1.4293 to 4.6118	0.0016
	∗3/∗3 (MH)	41 (24)	31 (18)	1.7786	1.0336 to 3.0607	0.0376
	∗1/∗3 (HE)+ ∗3/∗3 (MH); (HE + MH)	83 (49)	53 (31)	2.1061	1.3531 to 3.2781	0.0010
	∗1	216 (64)	256 (75)	1.000	—	—
	∗3	124 (36)	84 (25)	1.7496	1.2566 to 2.4358	0.0009

*Note:* HE, heterozygote.

Abbreviations: CI, confident interval; MH, mutant homozygote; NH, normal homozygote; OR, odds ratio.

The allele‐based analysis further supported the association between CYP3A5 variation and breast cancer susceptibility (Table 1). The frequency of the wild‐type allele (∗1) was higher among controls (75%) compared with patients (64%), whereas the mutant allele (∗3) was more prevalent in breast cancer cases (36%) than controls (25%). The CYP3A5∗3 allele was also markedly associated with the breast cancer risk (OR = 1.7496; 95% CI: 1.2566–2.4358; *p* = 0.0009). These results support a potential role of CYP3A53 variant in contributing to breast cancer susceptibility in Bangladeshi women.

### 3.4. Immune Infiltration Analysis of CYP3A5 Expression in Breast Cancer

To investigate the relationship between CYP3A5 expression and the tumor immune microenvironment, TIMER2.0 database analysis was performed using breast cancer (BRCA) sequencing data. Spearman correlation analysis demonstrated that CYP3A5 expression was significantly linked with immune‐cell infiltration levels of multiple immune‐cell and stromal‐cell populations. As shown in Figure 3, CYP3A5 expression exhibited a positive correlation with CD4^+^ T cells (Figure 3A) and CD8^+^ T cells (Figure 3B), suggesting a potential role in adaptive immune modulation. Similarly, innate immune components including mast cells (Figure 3C), neutrophils (Figure 3D), basophils (Figure 3H) and macrophages (Figure 3I) also showed positive correlations with CYP3A5 expression, indicating possible involvement in inflammatory regulation within the tumor microenvironment. Furthermore, stromal and structural components such as endothelial cells (Figure 3E), hematopoietic cells (Figure 3F), common lymphoid progenitor (Figure 3G), and cancer‐associated fibroblasts (Figure 3J) demonstrated a consistent positive association with CYP3A5 expression. Collectively, these results indicate that CYP3A5 expression may be linked with both immune infiltration and tumor microenvironment remodeling in breast cancer.

**FIGURE 3:**
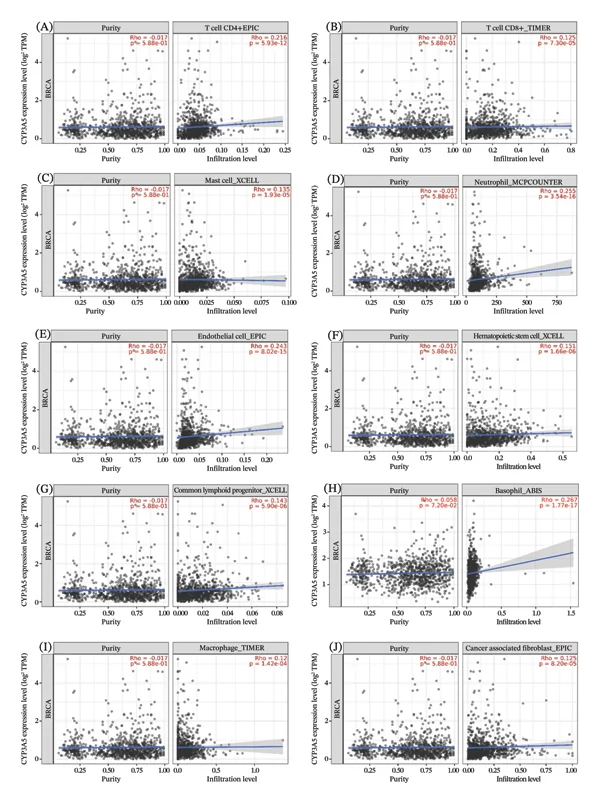
Association of CYP3A5 expression with immune‐cell infiltration in breast cancer. TIMER2.0‐derived Spearman correlation analyses showing the relationship between CYP3A5 transcript levels and estimated infiltration of CD4^+^ T cells (A), CD8^+^ T cells (B), mast cells (C), neutrophils (D), endothelial cells (E), hematopoietic cells (F), common lymphoid progenitors (G), basophils (H), macrophages (I), and cancer‐associated fibroblasts (J) across breast cancer transcriptomic datasets.

### 3.5. Association of CYP3A5 Expression With Survival Outcomes in Breast Cancer

To measure the prognostic significance of CYP3A5 expression, survival analysis was performed using TCGA breast cancer (BRCA) datasets accessed through the UALCAN portal. Kaplan–Meier survival curves were generated by stratifying patients according to CYP3A5 expression levels across different clinical and demographic subgroups.

As shown in Figure 4A, survival outcomes differed significantly among racial groups, with markedly reduced survival (*p* = 0.0012) observed in Caucasian patients exhibiting high CYP3A5 expression compared with other racial subgroups. Gender‐based stratification (Figure 4B) also revealed a significant survival disparity (*p* = 0.00018), where female patients with elevated CYP3A5 expression demonstrated poorer survival outcomes relative to other groups. When analyzed according to breast cancer subtype (Figure 4C), significant variation (*p* = 0.0026) in survival patterns was observed across tumor categories, with triple‐negative breast cancer patients showing notably lower survival rates in association with high CYP3A5 expression compared to other subtypes. Similarly, stratification by menopausal status (Figure 4D) indicated distinct survival differences between premenopausal and postmenopausal groups. Postmenopausal patients with high CYP3A5 expression exhibited reduced overall survival compared with other subgroups (*p* < 0.0001). Overall, these findings indicate that CYP3A5 expression is associated with differential survival outcomes across clinical subgroups, suggesting a potential prognostic relevance; however, its impact is not uniformly consistent across all demographic and clinical categories within TCGA breast cancer datasets.

**FIGURE 4:**
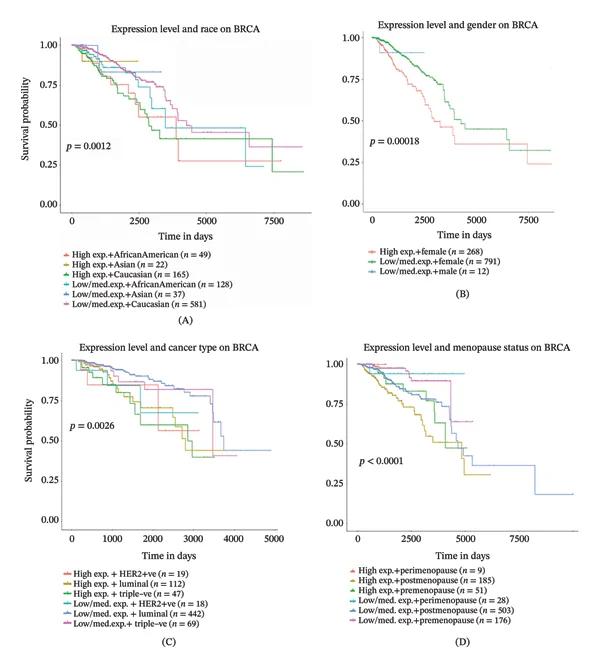
Prognostic association of CYP3A5 expression across clinical subgroups of breast cancer. Kaplan–Meier analyses of overall survival according to CYP3A5 expression using TCGA breast cancer data accessed through the UALCAN portal. Survival was evaluated across race (A), sex (B), breast cancer subtype (C), and menopausal status (D). Statistical differences were assessed using the log‐rank test. Significance is denoted as ns, not significant;  ^∗^ *p* < 0.05;  ^∗∗^ *p* < 0.01; and  ^∗∗∗^ *p* < 0.001. “Exp.” denotes CYP3A5 expression level, whereas “med.” denotes medium expression.

## 4. Discussion

Breast cancer represents a substantial health burden among Bangladeshi women, highlighting the need to identify genetic factors that may contribute to individual susceptibility. Accordingly, the study investigated the relationship between the CYP3A5∗3 genetic polymorphism and the risk of breast cancer.

The CYP3A5∗3 allele is a loss‐of‐function variant that makes another splice site, resulting in a truncated, nonfunctional enzyme and markedly reduced CYP3A5 expression. Individuals carrying this variant therefore exhibit decreased capacity for xenobiotic detoxification and steroid hormone metabolism, which may influence carcinogenesis pathways [7]. Despite being functionally inactive, CYP3A53 is highly prevalent globally. Its high frequency in Asian and Caucasian populations is thought to reflect neutral evolutionary selection, population bottlenecks, and genetic drift, rather than a deleterious selective disadvantage [19]. Reported frequencies exceed 70% in many Eurasian populations, whereas functional CYP3A5∗1 alleles are more common in African populations, demonstrating strong interethnic variation [7].

The results indicate that CYP3A5∗3 genotype variation is significantly associated with the risk of breast cancer. Compared with the wild‐type genotype (∗1/∗1), both the HE (∗1/∗3) and homozygous mutant (∗3/∗3) genotypes exhibited significantly higher risk of breast cancer. This suggests that reduced CYP3A5 enzymatic activity may impair detoxification of carcinogens and alter estrogen metabolism, thereby contributing to breast carcinogenesis [19, 20]. Comparable associations have been described in populations of Asian and European ancestry, providing further support for a potential contribution of CYP3A5 genetic variation to breast cancer susceptibility [7, 21, 22].

In addition to genetic susceptibility, multiple demographic and reproductive factors contribute to breast cancer risk. In our study, higher susceptibility was observed in women aged 36–45 years, consistent with the established role of cumulative hormonal exposure and age‐related genomic instability [23]. Reproductive factors such as nulliparity and reduced breastfeeding were also associated with increased risk, likely due to prolonged estrogen exposure and reduced breast epithelial differentiation [24]. Diabetes mellitus was another important risk factor, possibly through insulin resistance–mediated proliferative and antiapoptotic signaling pathways [25].

Sociodemographic factors such as education level and religion do not directly cause breast cancer biologically; however, they influence disease risk indirectly through healthcare access, awareness, reproductive behavior, lifestyle patterns, and screening practices. Lower educational attainment is associated with delayed diagnosis and reduced participation in screening programs, while cultural and religious practices may shape reproductive decisions, diet, and healthcare‐seeking behavior, thereby modifying population‐level risk.

Although CYP3A5∗3 is significantly associated with breast cancer risk, it cannot be considered an independent causal determinant. Breast cancer is a multifactorial disease, arising from complex interactions between genetic, environmental, hormonal, and lifestyle factors [26]. Therefore, CYP3A5∗3 should be interpreted as a susceptibility marker rather than a sole causative factor. Further studies incorporating gene–gene interactions, functional expression analysis, and environmental exposures are required to clarify its biological role.

The in silico immune infiltration analysis demonstrated that CYP3A5 transcript abundance was positively associated with the estimated presence of diverse immune and stromal populations within breast tumors, including CD4^+^ and CD8^+^ T cells, macrophages, neutrophils, mast cells, endothelial cells, hematopoietic cells, and cancer‐associated fibroblasts. Collectively, these observations raise the possibility that CYP3A5 expression is linked to the immune and stromal landscape of breast tumors, potentially reflecting a role in biological processes associated with tumor progression. Previous studies have shown that the composition of the tumor immune microenvironment plays a critical role in breast cancer development, therapeutic response, and clinical outcome. Therefore, altered CYP3A5 expression may reflect interactions between metabolic pathways and immune regulation within the tumor microenvironment. However, it should be emphasized that these observations were derived from the TCGA gene expression datasets and represent associations at the transcriptional level rather than evidence of a direct effect of the CYP3A5∗3 (rs776746) polymorphism on immune‐cell infiltration. Additional functional studies integrating genotype, gene expression, and immune profiling in the same patient cohort are required to elucidate the underlying biological mechanisms.

Consistent with our survival analysis findings, differential CYP3A5 expression appears to be associated with variability in overall survival across distinct clinical and demographic subgroups of breast cancer patients. Specifically, poorer survival observed in patients with high CYP3A5 expression—particularly among Caucasian, female, postmenopausal, and triple‐negative breast cancer subgroups—suggests a potential context‐dependent prognostic role of this gene. These findings may be explained by the involvement of CYP3A5 in xenobiotic metabolism and steroid hormone processing, which can influence tumor progression and treatment response [27, 28]. Previous studies have similarly reported that altered expression of CYP3A family enzymes may affect cancer outcomes through modulation of drug metabolism and tumor microenvironment interactions [29–31]. However, the inconsistency across subgroups in TCGA data indicates that CYP3A5 alone may not serve as a universal prognostic marker but rather functions within a broader network of genetic, hormonal, and environmental factors influencing breast cancer progression. Because the immune‐infiltration and survival analyses relied on independent public gene‐expression datasets rather than the genotyped Bangladeshi cohort, these findings provide contextual biological evidence and should not be interpreted as direct confirmation of the CYP3A5∗3–breast cancer association. Further studies integrating genotype, CYP3A5 expression, immune characteristics, and clinical outcomes within the same cohort will be important for establishing mechanistic and prognostic relationships.

## 5. Limitations

Several limitations should be acknowledged when interpreting these findings. Most importantly, the study included a relatively modest cohort of 170 breast cancer patients and 170 controls, which may have constrained statistical power for detecting smaller genetic effects and resulted in less precise effect estimates for some genotype comparisons. Consequently, the findings should be interpreted cautiously and may not be fully generalizable to the broader Bangladeshi population. Second, although PCR–RFLP is a well‐established genotyping method, more advanced techniques such as real‐time PCR or next‐generation sequencing could provide greater analytical accuracy.

Third, although several demographic and lifestyle factors were evaluated, important potential confounders, including detailed dietary habits, hormone receptor status (ER, PR, and HER2), and comprehensive family history, were not incorporated into multivariable‐adjusted analyses because complete data were unavailable for all participants. Therefore, residual confounding cannot be excluded.

Furthermore, the case–control genetic association study and the in silico analyses were conducted using independent datasets. The case–control study examined the association between CYP3A5∗3 genotypes and breast cancer risk, whereas the immune‐infiltration and survival analyses evaluated CYP3A5 gene expression using TCGA datasets. Since CYP3A5 expression was not measured in the genotyped participants, the present study cannot determine whether the CYP3A5∗3 polymorphism directly influences gene expression, immune‐cell infiltration, or clinical outcomes. Therefore, the in silico findings should be regarded as complementary evidence supporting the potential biological relevance of CYP3A5 rather than direct mechanistic validation of the observed genetic association.

Finally, the case–control design precludes causal inference. Future studies involving larger, multicenter cohorts with integrated genotyping, transcriptomic profiling, protein expression analysis, immune characterization, and detailed clinicopathological data are needed to independently validate the associations and elucidate the biological contribution of CYP3A5 to breast cancer susceptibility and disease progression.

## 6. Conclusion

In this Bangladeshi cohort, CYP3A5∗3 (rs776746) variation was associated with breast cancer susceptibility, highlighting its potential utility as a genetic risk indicator. Associations with reproductive, metabolic, and behavioral characteristics further emphasize the complex interplay of inherited and nongenetic determinants of disease. The complementary expression‐based analyses provide additional biological context for the potential involvement of CYP3A5 in the breast tumor microenvironment. Larger, multicenter studies incorporating genotype, molecular profiling, functional validation, and detailed experimental data are warranted to endorse the result and define the contribution of CYP3A5 to breast cancer initiation and progression.

## Author Contributions

Sm Faysal Bellah: conceptualization, methodology, formal analysis, writing–original draft, and supervision.

## Funding

No possible external financial support was received.

## Disclosure

The author has read and approved the final version of the manuscript. Sm Faysal Bellah had full access to all the data in this study and takes complete responsibility for the integrity of the data and the accuracy of the data analysis.

## Ethics Statement

The study protocol was approved by the ethical review committees of the participating hospitals (Approval No. BRCA‐2023‐32) and was conducted in accordance with the principles of the Declaration of Helsinki.

## Consent

Written informed consent was obtained from all participants before study enrollment. Participants were adequately informed about the aims and purposes of the research conducted and also advised of their voluntary contribution and their right to withdraw at any time. Consent records were securely maintained to protect participant confidentiality and privacy.

## Conflicts of Interest

The author declares no conflicts of interest.

## Data Availability

All data generated or analyzed during this study are included in this article.
